# Compound α-keto acid tablet supplementation alleviates chronic kidney disease progression via inhibition of the NF-kB and MAPK pathways

**DOI:** 10.1186/s12967-019-1856-9

**Published:** 2019-04-11

**Authors:** Meng Wang, Huzi Xu, Octavia Li-Sien Chong Lee Shin, Li Li, Hui Gao, Zhi Zhao, Fan Zhu, Han Zhu, Wangqun Liang, Kun Qian, Chunxiu Zhang, Rui Zeng, Hanjing Zhou, Ying Yao

**Affiliations:** 1 Department of Nephrology, Tongji Hospital, Tongji Medical College, Huazhong University of Science and Technology, 1095 Jiefang Ave, Wuhan, 430030 Hubei China; 2 Department of Nutrition and Food Hygiene, School of Public Health, Tongji Medical College, Huazhong University of Science and Technology, 1095 Jiefang Ave, Wuhan, 430030 Hubei China; 3 Department of Nutrition, Tongji Hospital, Tongji Medical College, Huazhong University of Science and Technology, 1095 Jiefang Ave, Wuhan, 430030 Hubei China; 4 Department of Nephrology, Jinhua Hospital of Zhejiang University, 365 Renmin East Ave, Jinhua, 321000 Zhejiang China

**Keywords:** Compound α-ketoacid tablets, Ischemia–reperfusion, Progression of chronic kidney disease, Renal function decline

## Abstract

**Background:**

Keto-analogues administration plays an important role in clinical chronic kidney disease (CKD) adjunctive therapy, however previous studies on their reno-protective effect mainly focused on kidney pathological changes induced by nephrectomy. This study was designed to explore the currently understudied alternative mechanisms by which compound α-ketoacid tablets (KA) influenced ischemia–reperfusion (IR) induced murine renal injury, and to probe the current status of KA administration on staving CKD progression in Chinese CKD patients at different stages.

**Methods:**

In animal experiment, IR surgery was performed to mimic progressive chronic kidney injury, while KA was administrated orally. For clinical research, a retrospective cohort study was conducted to delineate the usage and effects of KA on attenuating CKD exacerbation. End-point CKD event was defined as 50% reduction of initial estimated glomerular filtration rate (eGFR). Kaplan–Meier analysis and COX proportional hazard regression model were adopted to calculate the cumulative probability to reach the end-point and hazard ratio of renal function deterioration.

**Results:**

In animal study, KA presented a protective effect on IR induced renal injury and fibrosis by attenuating inflammatory infiltration and apoptosis via inhibition of nuclear factor-kappa B (NF-κB) and mitogen-activated protein kinase (MAPK) pathways. In clinical research, after adjusting basic demographic factors, patients at stages 4 and 5 in KA group presented a much delayed and slower incidence of eGFR decrease compared to those in No-KA group (hazard ratio (HR) = 0.115, 95% confidence interval (CI) 0.021–0.639, *p *= 0.0134), demonstrating a positive effect of KA on staving CKD progression.

**Conclusion:**

KA improved IR induced chronic renal injury and fibrosis, and seemed to be a prospective protective factor in end stage renal disease.

## Background

Chronic kidney disease (CKD) has become a crucial public healthcare issue on account of its progressive, irreversible pathological changes and complications including anemia, dyslipidemia and hyperparathyroidism. Previous studies have demonstrated that the metabolism of protein, which mainly comes from daily diet, is involved in most of these adverse pathological changes [1]. Thus, dietary intervention with restricted protein intake has been proposed as an extremely important therapeutic strategy to delay the progressive decline of renal function and the development of CKD.

As a simple low protein diet is prone to malnutrition, it is often implemented with other nutritional interventions, such as weight control, sodium diet, keto-analogues [2–4]. Keto-analogues contained appropriate proportioned essential amino acids to meet the needs of human synthesis while reducing the production of metabolic waste. The study of keto-analogues combined with protein restriction started since 1967, Richards etc. suggested that a supply of keto-analogues averted the harmful effect produced by the metabolisms of Sulphur and phosphorus contained in natural foods [5]. Despite some claims that a conjunctive use of α-ketoacids was superfluous in patients with renal insufficiency, more researchers confirmed that the combined use of keto-analogues with protein restriction reduced the metabolic wastes or toxicities in CKD patients [6–9]. Despite keto-analogues administration playing an important role in clinical CKD adjunctive therapy, research focusing on its basic mechanism involved in improving renal disease were quite few. Furthermore, nephrectomy was the most used kidney disease model in animal studies. In Zhang and Wang’s research, keto-analogues supplemented with low protein diet (LPD) were proved to inhibit the intrarenal renin-angiotensin-system to attenuate proteinuria, and regulate Wnt7a/Akt/p70S6K pathway and apoptotic system to improve muscle mass [10, 11]. In Dongtao Wang’s study, keto-analogues and LPD were observed to protect 5/6 nephrectomised rats by suppression of oxidative damage and mitochondrial dysfunction [12]. Another paper mentioned that keto-analogues and LPD increased the expression of Kruppel-like factor-15 (KLF15), a transcription factor proved to reduce cardiac fibrosis, thus reduced the severity of kidney disease in a remnant model [13]. In a diabetic nephropathy model, it seemed that keto-analogues delayed the progression of diabetic nephropathy via inhibition of oxidative stress [14]. However, the limitation of animal models caused the mechanical explorations to be incomplete and imperfect. More needs to be done to improve the mechanism of keto-analogues intervention in renal protection.

The renal ischemia-reperfusion (IR) injury model confers the advantage of observing renal repair after acute kidney injury while mimicking transient renal ischemia in clinical cases such as multiple organ failure and pre-transplant kidneys, thus offering insight into allograft survival after kidney transplant [15, 16]. Keto-analogues therapy with LPD provides sufficient essential amino acid intake without protein waste and excess metabolic toxin production and accumulation. Further understanding of its mechanism of action in CKD would be beneficial for expanding its applications.

In this study, we adopted IR model to explore the molecular mechanism of compound α-ketoacid tablets (KA) in renal disease from the angle of inflammation and apoptosis. KA supplementation inhibited nuclear factor-kappa B (NF-κB) and mitogen-activated protein kinase (MAPK) pathways, resulting in an alleviation of inflammation and apoptosis, thus staving the progression of CKD. The animal experiment gave further evidence to support the reno-protective effects of KA in CKD as seen in our clinical research.

## Methods

### Animals

Male C57BL/6 mice aged 2-month-old (weighing 25 g) were purchased from Beijing Huafukang Laboratory Animal Technology Co., Ltd, Beijing, China and were acclimated for 1 week. Mice were anaesthetized with 1% sodium pentobarbital (0.01 mL/g, Sigma, USA) by intraperitoneal injection. IR surgery: bilateral kidneys of mice were clamped in 26 min (Roboz Surgical Instrument Co, Germany). Renal blood flow was restored in a few seconds after murine artery clamp was removed. Whole surgeries were performed at 36.6 °C–37.2 °C using a temperature control machine (FHC, USA). Animals were divided into three groups: Sham, IR + Nacl, IR + KA, n = 4–6/group. Mice in sham group underwent the same surgery excluding clamping renal vessels. Mice in IR + Nacl group underwent with IR surgery and were treated with Nacl. Mice in IR + KA group were operated with IR surgery and treated with KA (FreseniusKabi, Germany). Both kidneys were harvested 28 days after IR surgery. All experiments were approved by the Animal Care and Use Committee of Tongji Hospital (IACUC Number: S851) and performed in accordance with NIH guidelines.

### KA and LPD preparation and administration

Compound α-ketoacid tablets were confected by mixture of 0.375 g α-ketoacid to 6 mL 0.9% Nacl, to a final concentration of 62.5 mg/mL and were administered by gastric gavage. Administration concentration was 1000 mg per kg mouse weight per day. LPD was supplied by WQJX BIO-Technology (Wuhan, China). The diet was composed of casein (6.5%), starch (66.45%), glucose (10%), soybean oil (7%), fibrin (5%), mineral salt (3.5%), vitamin (1%), l-cystine (0.3%) and choline chloride (0.25%) in accordance with rodent diet of American Dietetic Association, with a calorie of 3.5 kcal/g to meet energy need. In IR + KA group, mice were treated with KA and LPD. In comparison, mice in the IR + Nacl group were treated with 0.9% Nacl and LPD.

### BUN, TC and TG detection

Blood urea nitrogen (BUN), total cholesterol (TC) and triglyceride (TG) were tested using kits (Changchunhuili, China) according to the manufacturer’s instructions.

### Histological, immunocytochemical and immunofluorescent staining

Bilateral kidneys of mice were fixed in 4% paraformaldehyde and embedded in paraffin. The paraffin-embedded kidneys were sectioned at 3 μm. Pathological staining for Periodic Acid-Schiff staining (PAS), and Sirius red were performed to evaluate renal tubular injury grade and interstitial fibrosis. For immunofluorescent (IF) staining, sections were stained with primary antibody Kim-1 (1:800; R&D), lotus tetragonolobus lectin (LTL, 1:100, Vector Lab), alpha-smooth muscle actin (α-SMA, 1:100, Abcam), Collagen I (1:100, Abcam), CD45 (1:50, Biolegend), CD3 (1:50, Guge, Wuhan) and Ly6G (1:50, Biolegend) at 4 °C overnight and fluorescent labeled secondary antibodies. Nuclei were stained with 4′-6-diamidino-2-phenylindole (DAPI). Data was analyzed by Image Pro Plus software (Media Cybemetics, Rockville, MD, USA) in a blinded manner.

### Western blot

Renal tissues were lysed in RIPA lysis buffer (Promoter, Wuhan, China) containing protease inhibitor (Promoter, Wuhan, China). Cell proteins were collected in the same way. Total protein concentration was determined using a BCA protein assay kit (Promoter, Wuhan, China). Proteins were separated by sodium dodecyl sulfate–polyacrylamide gel electrophoresis (SDS-PAGE) and transferred to polyvinylidene difluoride membranes (PVDF, Millipore, Billerica, MA, USA). The membranes were blocked with 5% skimmed milk in TBS with 0.1% Tween-20 for 1 h at 37 °C and then probed with antibodies against α-SMA (1:5000, Abcam), PDGFR-β (1:3000, Abcam), Fibronectin (1:1000, Abcam), Bcl-2 (1:1000, Abclonal), BAX (1:1000, Abclonal), Caspase 3 (1:1000, Abclonal), NFATc-1 (1:1000, Abcam), total and phosphorylated-65, -38 and -ERK (1:1000, cell signaling technology, USA) and GAPDH (1:4000, Abbkine) at 4 °C overnight. The blots were washed next day and incubated with HRP-conjugated secondary antibodies for 1 h at 37 °C. The signal intensities of targeted band were quantified using Image J (NIH, USA).

### Quantitative real-time PCR

Total RNA was extracted using Trizol reagent according to the manufacturer’s instructions (Invitrogen, USA). One microgram RNA was reverse transcribed into first strand cDNA using the GoScript reverse transcription system (Promega, USA) in a 20 μL reaction system. The cycling parameters were used as follow: 40 cycle pf denaturation at 95 °C for 15 s and annealing at 60 °C for 60 s. Triplicated experiments for each sample were processed. Quantitative PCR was conducted using SYBR master mix (Qiagen, Germany) on the Roche light 480II. The mRNA expression levels of several markers, including TGF-β, fibronectin and Collagen I were conducted via the comparative cycle threshold (Ct) method and normalized to the expression levels of GAPDH. Primer sequences were listed in Table 1.

**Table 1:** Primer sequences for qRT-PCR [52]

Genes	Sequences
GAPDH	Forward primer: 5′-TTGATGGCAACAATCTCCAC-3′ Reverse primer: 3′-CGTCCCGTAGACAAAATGGT-5′
TGF-β	Forward primer: 5′-CTTCAATACGTCAGACATTCGGG-3′ Reverse primer: 3′-GTAACGCCAGGAATTGTTGCTA-5′
Fibronectin	Forward primer: 5′-GCTCAGCAAATCGTGCAGC-3′ Reverse primer: 3′-CTAGGTAGGTCCGTTCCCACT-5′
Collagen I	Forward primer: 5′-ATGGATTCCCGTTCGAGTACG-3′ Reverse primer: 3′-TCAGCTGGATAGCGACATCG-5′

### Basic information of clinical CKD patients collected from Tongji Hospital

A total number of 894 CKD outpatients and inpatients were recruited from April 2015 to September 2018 in Tongji hospital, Tongji medical college, Huazhong university of science and technology for cross-sectional and retrospective study. A total number of 452 patients were excluded, (1) hemodialysis, peritoneal dialysis or renal transplantation at the beginning of enrolled time; (2) the follow-up time was less than 2 times; (3) basic data substantially missing. Among the remaining enrolled 442 patients, 119 were at stage 1 and 2, only 9 patients were prescribed KA, which greatly influenced the result of analysis. For the remaining 323 patients at stage 3 to 5, 148 patients took KA were divided into KA group, the other 175 patients were divided into No-KA group. The clinical study was approved by Medical Ethics Committee of Tongji Hospital, Tongji medical college, Huazhong University of Science and Technology (TJ-IRB20180501). We registered in Chinese Clinical Trial Registry (ChiCTR1800016536).

### Statistical analysis

Data was expressed as count value (%) for categorical variables and mean ± SD for continuous variables. Comparisons between the two groups were made by nonparametric *T* test for continuous variables while χ^2^ test for categorical variables. Kaplan–Meier analysis and COX proportional hazard regression model were adopted to calculate the cumulative probability to reach the end-point and hazard ratio of renal function deterioration. The statistical analyses were performed by SAS v9.4 (SAS, Inc., 200 Cary, NC, USA), and all P-values calculated as two-sided. The association was considered significant with p-values less than 0.05.

## Results

### KA supplement protected mice from IR-induced renal injury and metabolic disorders

**Fig. 1:**
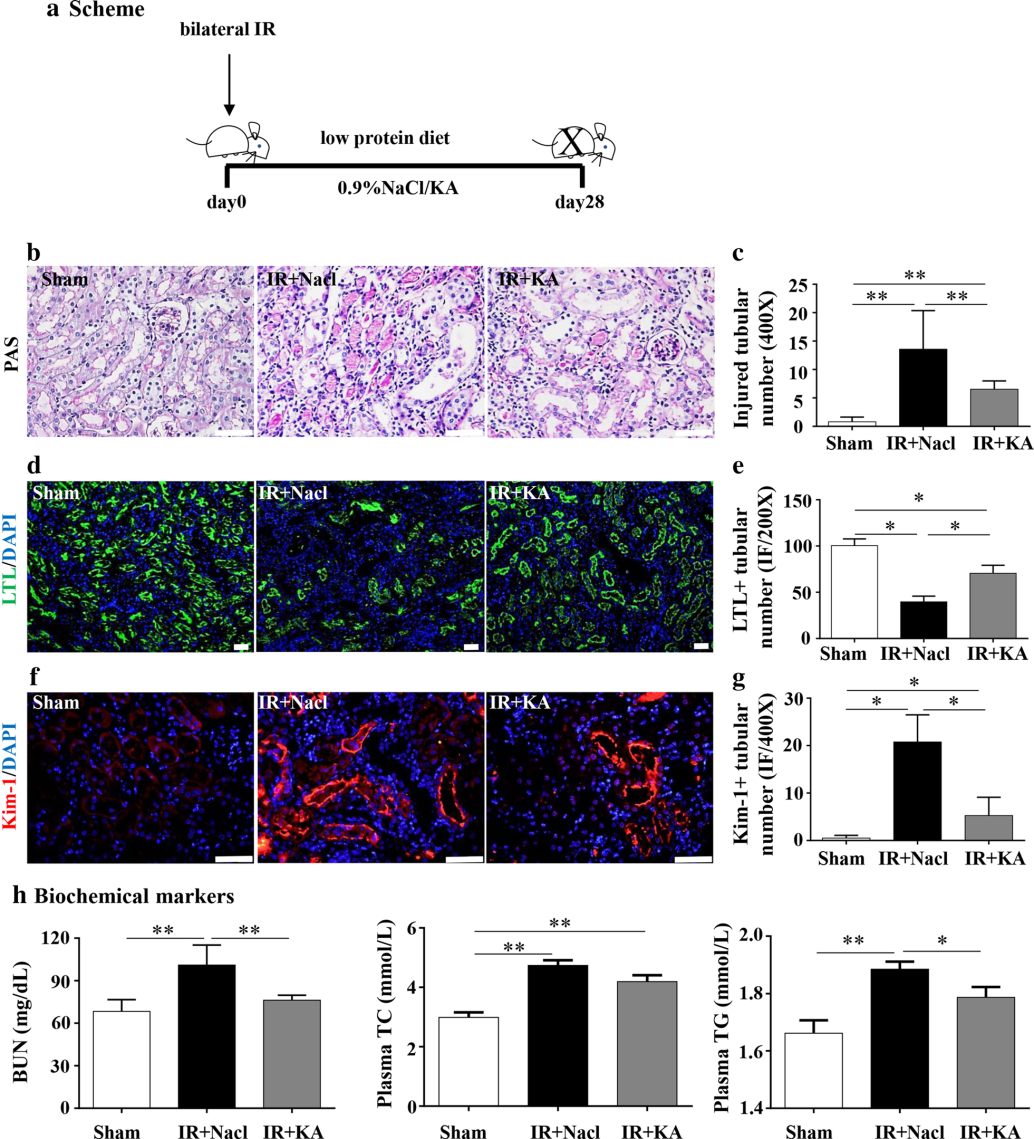
KA supplement protected mice from IR induced renal injury and metabolic disorders. **a** The experiment scheme in vivo. Mice were divided into 3 groups: Sham, IR + Nacl (IR surgery + 0.9% Nacl solvent), IR + KA (IR + KA dissolved in 0.9% Nacl: 1000 mg/kg/d). LPD was administrated after IR surgery, at the same time with KA for 28 days. **b**, **c** PAS staining showed a mass of tubular expansions, casts and loss of tubular brush border in IR + Nacl group. With the treatment of KA, tubular expansions were ameliorated and casts were reduced. **d**, **e** LTL staining showed IR caused more than 2.0-folds decrease of healthy tubules, KA preserved nearly 1.8 folds increased remnant healthy tubules compared to IR + Nacl group. **f**, **g** The detection of Kim-1 showed a same phenomenon as LTL. An extensive injure renal tubules were founded in IR + Nacl group. In IR + KA group, the number of kim-1 positive tubules decreased. **h** Serum BUN and plasma TC and TG were detected by using kits. KA reduced IR induced enhancement of BUN and TG. Otherwise, KA showed a slight decrease in IR induced TC disorder, the concentration of TC in IR + Nacl was 4.74 mmol/L while in IR + KA was 4.19 mmol/L. Scale bars: 50 μm. Data were presented as mean ± SD, *p < 0.05, **p < 0.01 (unpaired nonparametric t-test); n = 4–6/group

To better explore the effect of KA on mice, we adopted oral administration of KA for 28 days after bilateral IR surgery (Fig. 1a). A basic pathological PAS staining showed that IR damaged renal tubules and caused massive cast formations, expansions and necrosis (Fig. 1b). LTL, short for Lotus tetragonolobus lectin, which combines specifically with proximal tubular brush borders, is a marker used for healthy renal tubules. LTL staining showed that after IR surgery, few remaining LTL positive healthy tubules existed (Fig. 1d). Staining of the other marker Kim-1, an abbreviation for Kidney Injury Molecule-1, which is specifically dyed in injured tubules, displayed a similar image to LTL: the number of injured tubules increased as the number of healthy tubules decreased (Fig. 1f). In IR + KA group, the mice treated with KA presented an improved state of operated kidneys. The number of cast and tubular expansions were conspicuously reduced (Fig. 1b, c). Nearly twice as many healthy renal tubules were preserved after KA treatment compared to the IR + Nacl group, only sparse tubules were marked by Kim-1 (Fig. 1d–g). Next, we measured biochemical markers and found that KA can not only improve IR-induced deterioration of renal function, but also remit IR-induced lipid disorders. TG levels dropped significantly in IR + KA group. Otherwise, KA caused a slight but statistically insignificant reduction on cholesterol (Fig. 1h). The above results implied an additional positive regulation of lipid metabolism brought by KA, aside from a fundamental therapeutic effect on renal injury.

### KA supplement improved IR-induced interstitial fibrosis

**Fig. 2:**
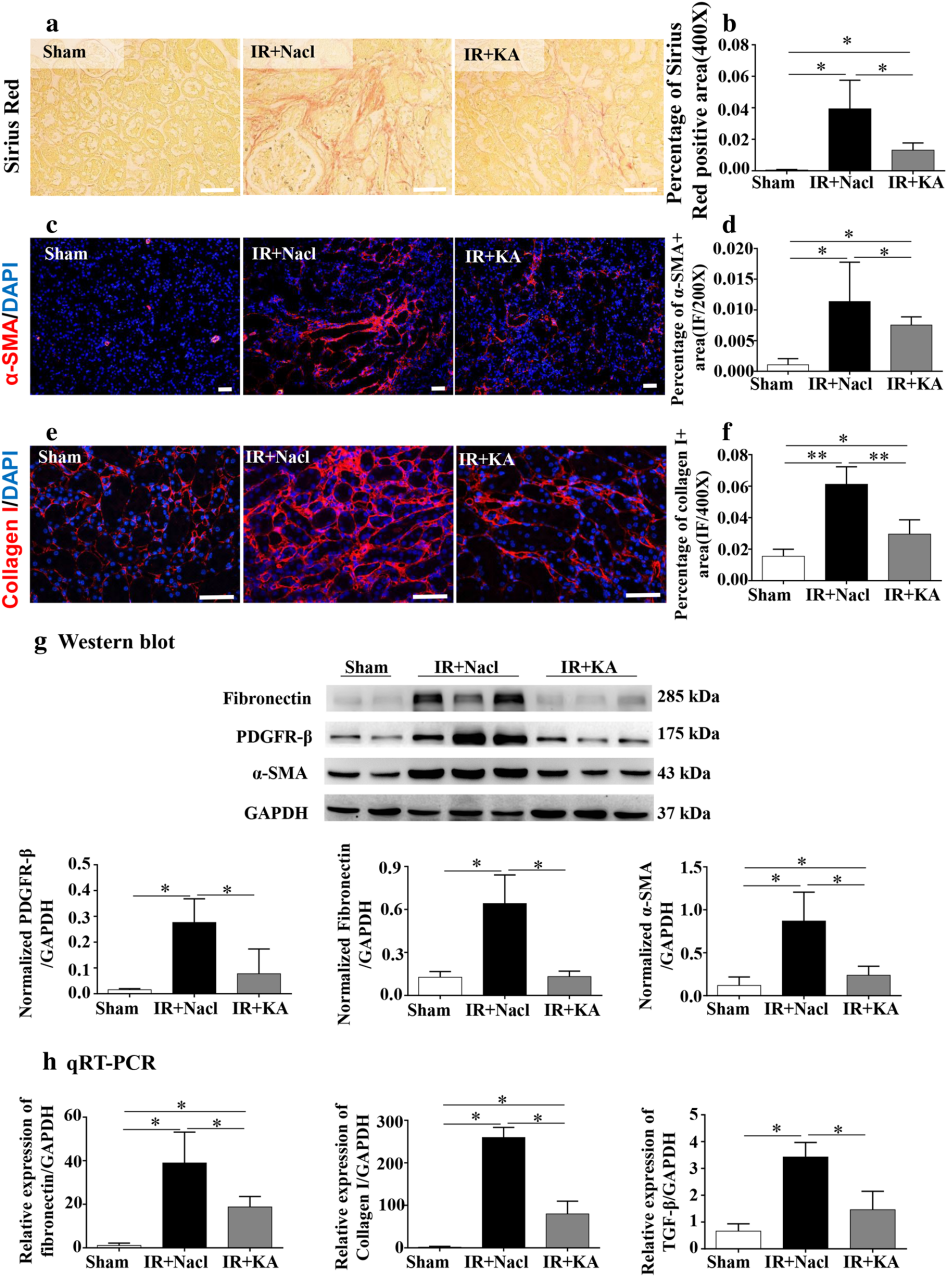
KA supplemented with LPD improved IR induced interstitial fibrosis. **a**–**f** Pathological staining Sirius red (red), myofibroblast marker α-SMA (red) and collagen I (red) staining presented increased interstitial myofibroblast and collagen deposition in IR + Nacl group. KA improved both myofibroblasts and collagen. Renal interstitial fibrosis was improved in IR + KA group. **g**, **h** The detections of fibrosis related factors: PDGFR-β, fibronectin, collagen I and TGF-β showed KA had an improved effect on IR induced renal fibrosis in protein and/or RNA levels. Scale bar: 50 μm. Data were presented as mean ± SD. *p < 0.05, **p < 0.01 (unpaired nonparametric t-test); n = 3–5/group

As renal fibrosis is the end-way of all chronic kidney disease, a measurement of fibrosis was necessary to evaluate the therapeutic efficiency of KA in kidney. Sirius red staining, a pathological staining to test collagen deposition showed broad collagen fibers were generated and accumulated in renal interstitium after IR. In IR + KA group, only filar collagen appeared in interstitium (Fig. 2a, b). Immunofluorescence labeling of alpha-Smooth muscle actin (α-SMA, a marker of myofibroblast) and collagen I demonstrated that IR led to a serious amount of myofibroblasts and collagen production in extracellular interstitium. With the administration of KA, the distribution of α-SMA and collagen I became sparse, the accumulation of myofibroblasts and collagen was distributed less densely (Fig. 2c–f). Next, we detected fibrosis markers in protein and RNA levels. KA significantly reduced the expressions of α-SMA, collagen I, Fibronectin and platelet-derived growth factor receptor (PDGFR-β), a marker of pericytes, which was demonstrated to be another source of fibrosis (Fig. 2g, h). A fibrosis related factor, transforming growth factor β (TGF-β), mainly participating in transforming fibroblasts into myofibroblasts and regulating fibrosis, was expressed abundantly in IR + Nacl group, with a greater than fivefold increase in RNA level compared to Sham group. The use of KA could inhibit the expression of TGF-β (Fig. 2H). The above data implied that KA supplement could improve IR induced renal injury while simultaneously slowing down progression to renal interstitial fibrosis.

### KA supplement attenuated IR-induced inflammatory infiltration and apoptosis

**Fig. 3:**
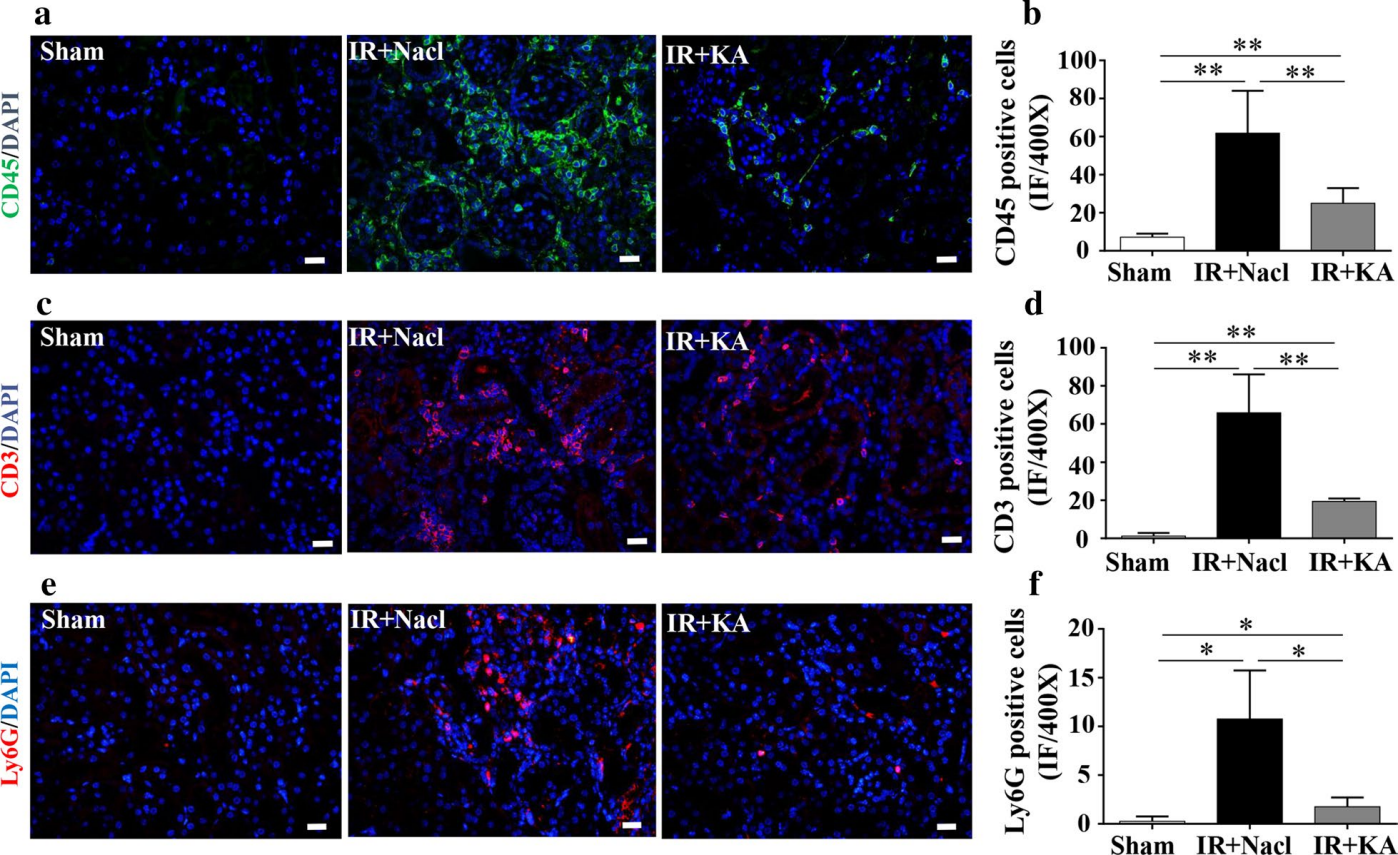
KA supplemented with LPD attenuated IR induced inflammatory infiltration. **a**–**f** CD45 (green), CD3 (red), Ly6G (red) and DAPI (blue) staining presented a more than 8.0-folds of CD45 positive T cell, 60-folds of CD3 positive T cells and 40 folds of Ly6G positive neutrophils in IR + Nacl group after IR surgery. KA improved mature T cell and neutrophil granulocytes infiltration. Scale bar: 20 μm. Data were presented as mean ± SD. *p < 0.05, **p < 0.01 (unpaired nonparametric t-test); n = 4–5/group

**Fig. 4:**
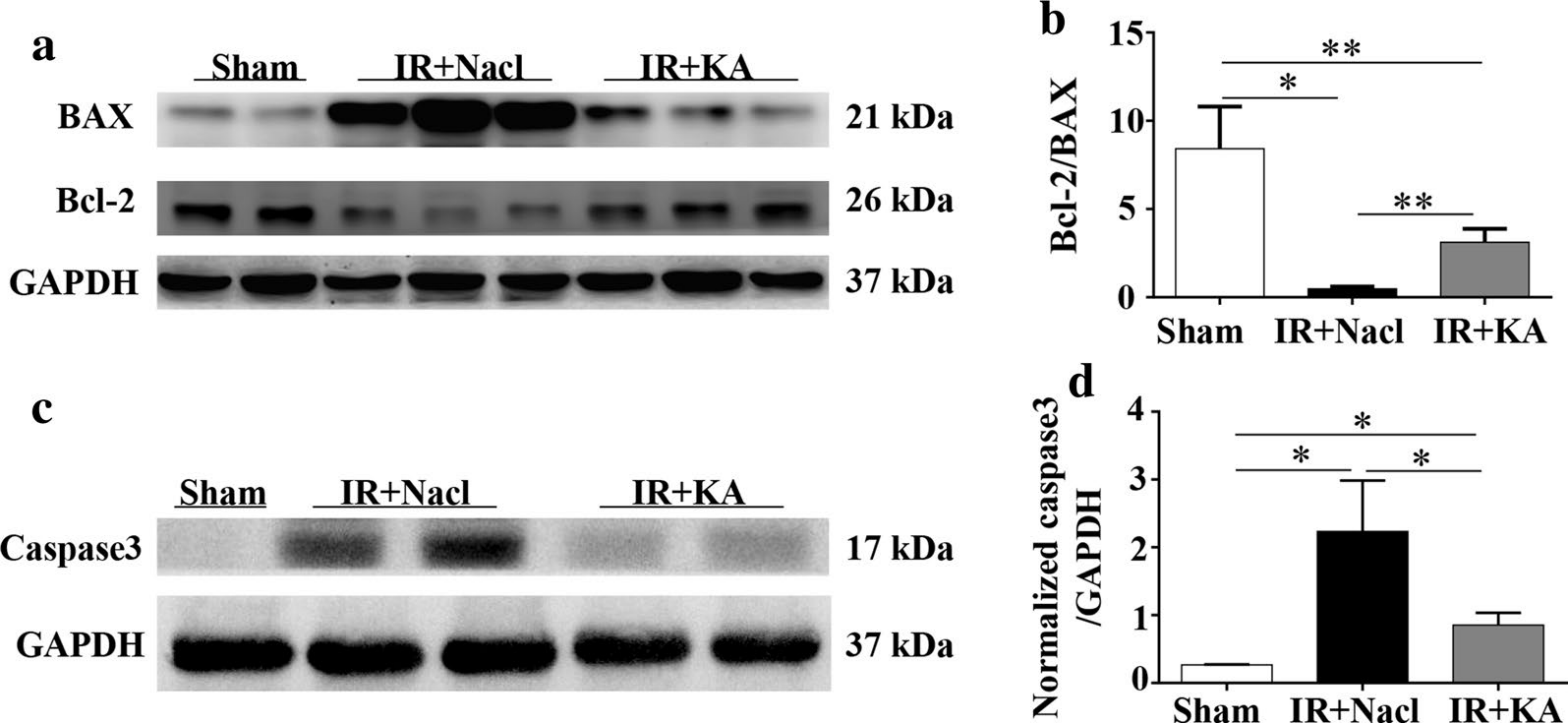
KA attenuated IR induced apoptosis. **a**–**d** Western blot analysis and histograms of apoptosis markers: Bcl-2, BAX and caspase3. After IR surgery, the apoptosis related factors BAX and Caspase 3 increased. KA reduced the expressions of BAX and Caspase 3, without improved the anti-apoptotic factor Bcl-2. However, the ratio of Bcl-2/BAX increased in IR + KA group, meaning a anti-apoptotic effect of KA on IR. Data were presented as mean ± SD. *p < 0.05, **p < 0.01 (unpaired nonparametric t-test); n = 3–5/group

In IR injury, renal injury and apoptosis are regulated by some cytokines such as TGF-β, which is produced and secreted by leukocyte lineage cells, including lymphocytes, macrophages and dendritic cells. A chronic inflammatory condition promotes the increased production and activation of latent TGF-β, modulating the proliferation of fibroblasts and excessive deposition of extracellular matrix [17–19]. To further explore the related mechanisms of IR-induced fibrosis and a probable participating role of KA, we tested CD45, a marker presenting the whole T cells, CD3 for mature T cells and Ly6G for neutrophils. In day 28 of IR, there were still a large amount of inflammatory cells infiltration in injured kidney. The expressions of CD45 positive T cells and CD3 positive mature T cells increased widely in IR + Nacl group. With the treatment of KA, the area of inflammatory infiltration decreased more than 50% in IR + KA group (Fig. 3a-f). Later we detected the expressions of apoptotic factor BAX and Bcl-2, an anti-apoptotic marker. The ratio of Bcl-2/BAX decreased in IR + Nacl group, which meant increased apoptosis in operated kidney. Another apoptotic marker Caspase3 was also found to be increased in IR + Nacl. KA administration presented an anti-apoptotic effect on IR-induced renal injury, with an enhanced expression of Bcl-2/BAX and a reduction of Caspase3 in protein level (Fig. 4a-d).

### KA supplement might attenuate IR-induced inflammation and apoptosis by inhibiting the activation of NF-κB and MAPK pathways

**Fig. 5:**
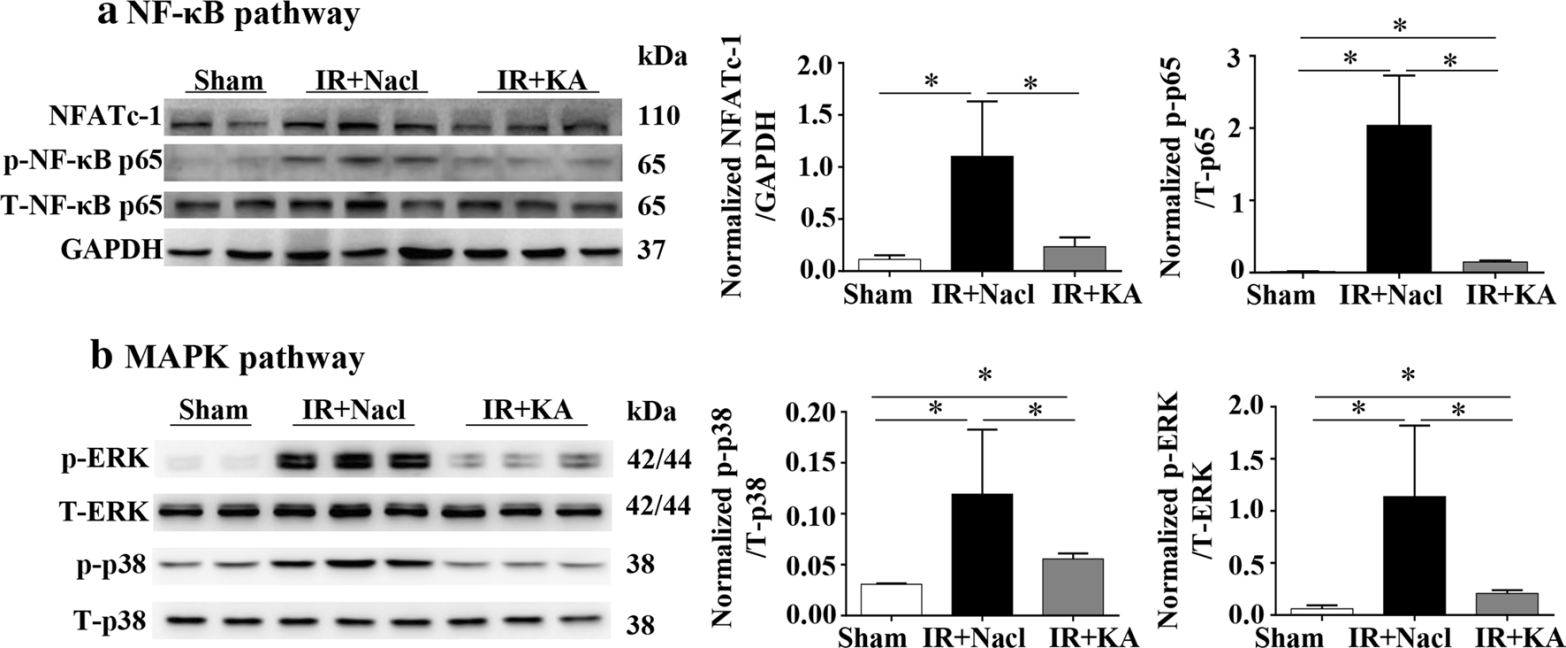
KA supplemented with LPD might attenuate IR induced inflammation and apoptosis by inhibiting the activation of NF-κB and MAPK pathways. **a** Western blot analysis and histograms of NF-κB pathway: p-p65 raised dramatically in IR + Nacl group compared to the Sham group. Its targeted molecule NFATc-1 increased after IR surgery too. KA inhibited the activation of NF-κB pathway, as well as NFATc-1. **b** MAPK pathway (p-p38 and p-ERK). KA presented a similar effect on MAPK pathway as NF-κB pathway. A nearly 2.0-folds increase of p-p38 was detected in IR + Nacl group compared to the Sham group. Half was decreased after the usage of KA. The expression of p-ERK was also reduced in protein level in KA group after IR. Data were presented as mean ± SD. *p < 0.05 (unpaired nonparametric t-test); n = 3/group

NF-κB and MAPK pathways are two classic signaling pathways participating in inflammation and apoptosis [20–24]. In IR + Nacl group, the expression of phosphorylated-p65 increased, as well as that of its downstream molecule NFATc-1, showing that IR promoted the activation of NF-κB pathway. With the treatment of KA, the activation of phosphorylated-p65 was partly inhibited, and the expression of NFATc-1 was reduced (Fig. 5a). At the same time, we measured MAPK pathway-related molecules and observed a similar increase of phosphorylated ERK and p38 in IR + Nacl group. In the IR + KA group, the activation was inhibited with a decrease of 50% in p-p38 and 80% in p-ERK compared to IR + Nacl group (Fig. 5b). Our results suggest that KA supplement alleviate renal inflammation and apoptosis via an inhibited activation of NF-κB and MAPK pathways.

### Basic demographic characteristics of clinical CKD patients at different stages and difference in renal function status between people with and without KA intervention

**Fig. 6:**
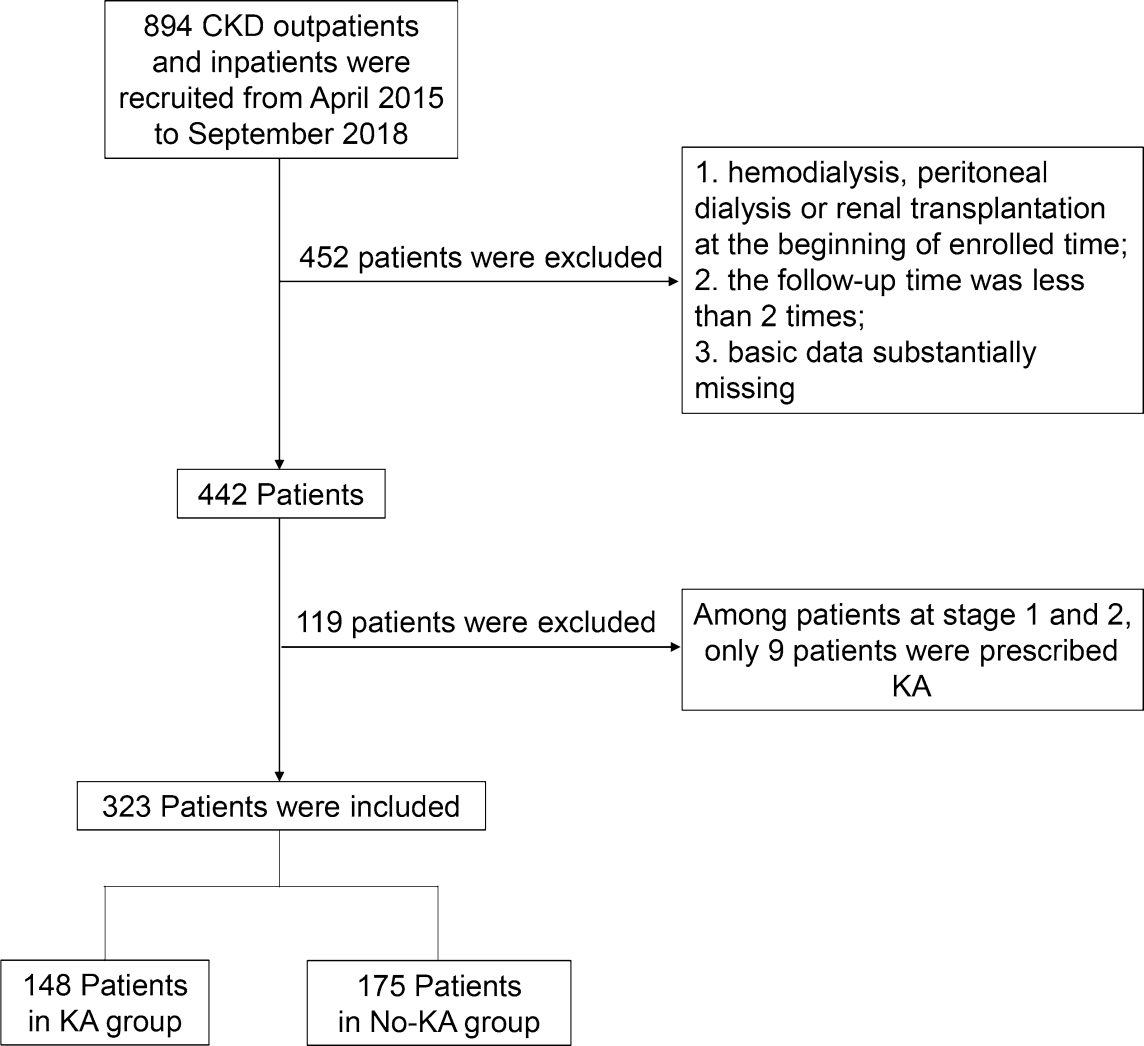
Patients flow diagram throughout study

The above research pointed to a protective effect of KA on IR induced chronic kidney injury in mice. Next, we collected basic information of patients to further explore the characteristic difference between patients with or without KA intervention (Fig. 6). Commonly, patients prescribed with KA were mainly at CKD stages 3 to 5, with 78/201 in the stage 3 group and 70/122 in the stage 4 and 5 groups (Table 2), and only 9 among 119 patients at CKD stages 1 and 2. Basic demographic characteristics showed an interesting phenomenon that CKD patients at stages 3 to 5 mainly possessed secondary level or higher education, and were engaged in intellectual fields of work or retired. Otherwise, the age of CKD patients at stage 3 to 5 peaked between 35 to 60, displaying a characteristic chronic progression feature of CKD. Next, we analyzed the renal function indexes of patients at stages 3 to 5, including eGFR, Scr, BUN and UA. In the group of patients at stage 3, a significant worsening of baseline renal function indexes was observed in people with KA, *p *= 0.000 for eGFR and Scr, *p *= 0.068 for UA. In end-point CKD stage groups, eGFR and Scr scores in KA group were still poorer compared to No-KA group (with *p *= 0.000 for eGFR and *p *= 0.009 for Scr). BUN in the KA group was higher than No-KA group (*p *= 0.053) at a value of about 9.2 ± 3.9 mmol/L. Combining patients at stages 3 to 5, a similar statistical difference was analyzed between KA and No-KA group, patients with KA prescription usually had worse renal conditions. However, taking only patients at stages 4 and 5 into account, no significant difference was detected in baseline eGFR, Scr, BUN, UA and ${\text{HCO}}_{3}^{ - }$ when comparing the KA with No-KA group (Table 3).

**Table 2:** Basic demographic characteristics of enrolled CKD patients

	No-KA	KA	*p* value
	(n = 175)	(n = 148)	
*CKD stage*			
3	123 (0.703)	78 (0.527)	*0.000*
4	42 (0.240)	42 (0.284)	
5	10 (0.057)	28 (0.189)	
*Gender*			
Man	108 (0.62)	78 (0.52)	0.114
Woman	67 (0.38)	70 (0.48)	
Age (year)	46.7 ± 12.6	46.0 ± 11.7	0.601
*Education*			
Primary	26 (0.15)	25 (0.17)	*0.030*
Secondary	96 (0.55)	97 (0.66)	
Higher	53 (0.30)	26 (0.16)	
*Occupation*			
Manual worker	17 (0.10)	30 (0.20)	*0.021*
Brain-worker	77 (0.44)	52 (0.35)	
Unemployed/retiree	81 (0.46)	66 (0.45)	
*Complication*			
No	119 (0.68)	101 (0.68)	1.000
Yes	56 (0.32)	47 (0.32)	
*Smoke*			
No	113 (0.65)	97 (0.66)	0.793
Sometimes	5 (0.03)	3 (0.02)	
Yes	57 (0.33)	43 (0.29)	
*Alcohol*			
No	123 (0.70)	107 (0.72)	0.381
Yes	52 (0.30)	36 (0.24)	

Italic values indicate significance of *p* value (*p* < 0.05)

**Table 3:** Baseline clinical characteristics of enrolled CKD patients

Renal function	CKD (3 to 5 stages)			CKD (4 and 5 stages)			CKD (3 stage)		
	No-KA (n = 175)	KA (n = 148)	*p* value*	No-KA (n = 52)	KA (n = 70)	*p* value*	No-KA (n = 123)	KA (n = 78)	*p* value*
*eGFR (mL/min/1.73* *m*^*2*^*)*									
Baseline	38.7 ± 14.1	30.7 ± 13.1	*0.000*	20.7 ± 6.2	19.1 ± 6.8	0.187	46.4 ± 8.4	41.1 ± 7.4	*0.000*
End-point	43.3 ± 20.7	31.4 ± 17.5	*0.000*	21.8 ± 11.5	19.4 ± 13.0	0.295	52.4 ± 16.5	42.1 ± 13.5	*0.000*
*Scr (μmol/L)*									
Baseline	187.0 ± 98.1	226.4 ± 107.7	*0.001*	296.7 ± 116.2	305.5 ± 108.4	0.669	140.6 ± 28.2	155.3 ± 28.4	*0.000*
End-point	186.3 ± 116.2	246.1 ± 136.8	*0.000*	298.1 ± 130.2	337.6 ± 140.2	0.115	139.0 ± 67.8	163.9 ± 60.1	*0.009*
*BUN (mmol/L)*									
Baseline	10.2 ± 5.2	11.9 ± 5.6	*0.008*	15.4 ± 6.2	15.6 ± 5.6	0.854	8.0 ± 2.5	8.5 ± 2.7	0.233
End-point	10.1 ± 5.4	13.8 ± 8.0	*0.000*	14.7 ± 5.8	19.0 ± 8.3	*0.002*	8.1 ± 3.7	9.2 ± 3.9	0.053
*UA (μmol/L)*									
Baseline	412.7 ± 113.4	425.3 ± 100.1	0.293	437.6 ± 114.9	422.2 ± 112.6	0.462	402.1 ± 111.5	428.1 ± 88.0	0.068
End-point	383.8 ± 89.1	373.8 ± 83.8	0.307	399.8 ± 105.0	357.3 ± 94.2	*0.023*	377.0 ± 81.0	388.9 ± 70.3	0.289
$HCO_{3}^{ - }$ *(mmol/L)*									
Baseline	22.5 ± 3.3	22.3 ± 3.1	0.546	20.7 ± 3.0	21.0 ± 2.9	0.493	23.2 ± 3.1	23.4 ± 2.8	0.762
End-point	24.1 ± 2.7	22.9 ± 2.7	*0.000*	22.8 ± 2.5	21.8 ± 2.2	*0.018*	24.6 ± 2.6	23.9 ± 2.6	0.054

Italic values indicate significance of *p* value (*p* < 0.05)

* Presented as comparison of the No-KA group with the KA group (t-test)

### The effects of KA on renal survival probability in CKD patients at different stages

**Fig. 7:**
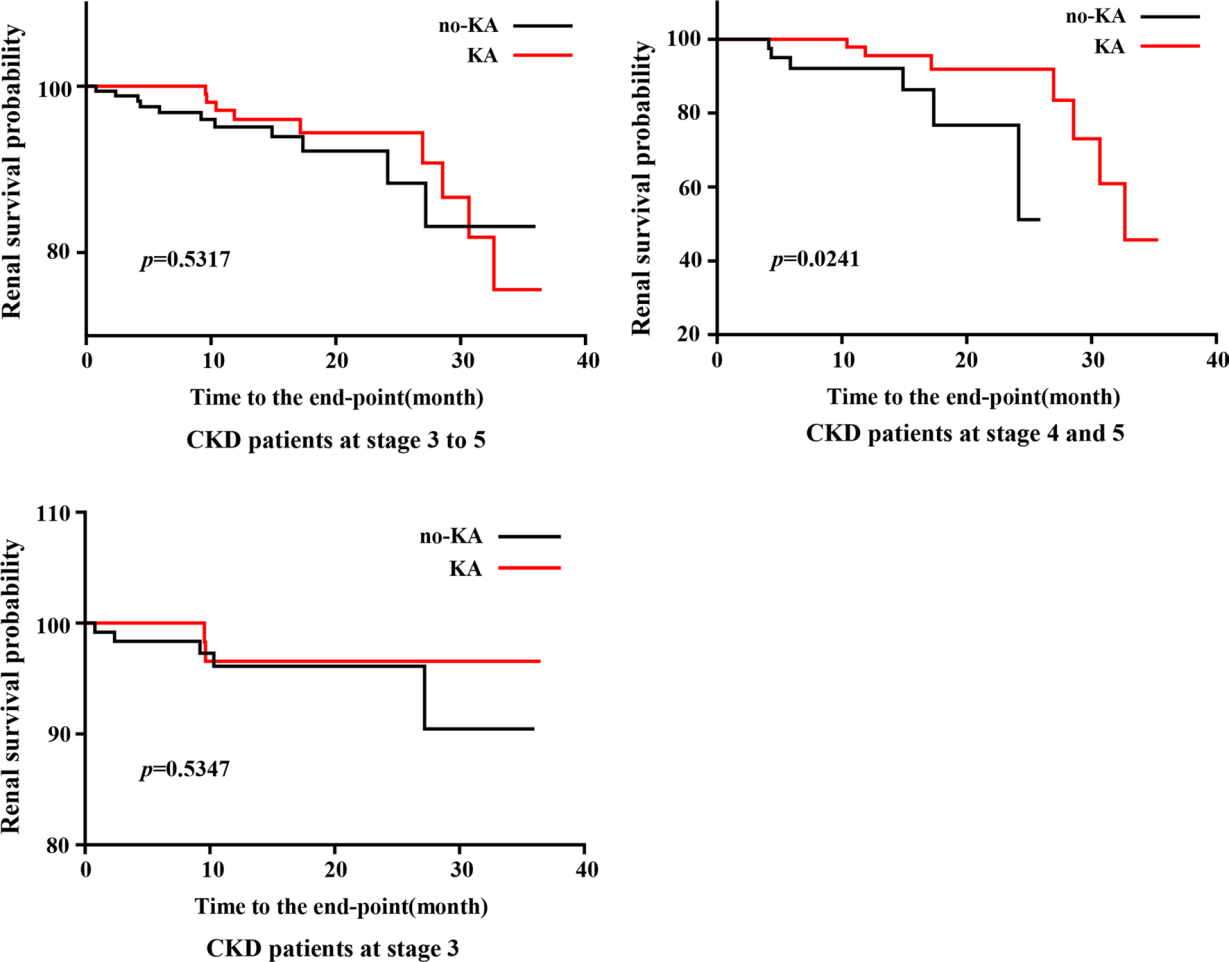
The effect of KA on renal survival probability in CKD patients at different stage. The probability to reach the end-point was lower in KA group than no-KA group at CKD stages 4 and 5 when adjusted in a log rank test (*p *= 0.0241). For patients at stage 3 to 5, KA also showed a protective effect on renal function before 30 months, after 30 months, patients in KA group presented a deteriorate renal situation. For patients at stage 3, the incidence of end-point (n = 0.035, 7/201) in KA group was lower than no-KA group, however, the whole incidence of end-point was rather less than at stage 4 and 5 (n = 0.107, 13/12)

We divided enrolled patients at CKD stages 3 to 5 into KA and No-KA groups according to their medications. An end-point event was defined as a > 50% reduction in the initial eGFR. In a Kaplan–Meier analysis, the cumulative probability to reach the end-point was lower in the KA group than the No-KA group (*p *= 0.0241) among patients at stages 4 and 5. For patients at stages 3 to 5, KA also showed a protective effect on renal function before the 30-month mark; after 30 months of treatment, patients in the KA group presented a deterioration in renal condition. Only taking patients at stage 3 into account, the incidence of end-point CKD events in the KA group was lower than the No-KA group. However, the total incidence of end-point events (n = 0.035, 7 end-point events among 201 patients) was lower than at stages 4 and 5 (n = 0.107, 13 among 122) (Fig. 7). After adjustment for basic demographic factors (including gender, age, complication, education, occupation, smoking and alcohol consumption history), people in the KA group presented a greatly delayed and slower incidence of eGFR decrease compared to the No-KA group [adjusted hazard ratio, 0.115; 95% CI 0.021 to 0.639; *p *= 0.0134 (Table 4)] among patients at stages 4 and 5, exhibiting a positive effect of KA on CKD progression.

**Table 4:** Adjust of demographic factors for CKD

Variate	CKD (3 to 5 stages)		CKD (4 and 5 stages*)*		CKD (3 stage)	
	HR (95% CI)	*p*	HR (95% CI)	*p*	HR (95% CI)	*p*
KA	0.701 (0.276–1.783)	0.4556	0.115 (0.021–0.639)	*0.0134*	0.490 (0.086–2.806)	0.4230
Gender	1.155 (0.324–4.122)	0.8243	1.631 (0.174–15.291)	0.6684	0.807 (0.069–9.486)	0.8645
Age	0.958 (0.919–0.999)	0.0456	0.914 (0.856–0.976)	*0.0069*	0.988 (0.926–1.055)	0.7200
Complication	0.394 (0.109–1.421)	0.1547	0.272 (0.032–2.318)	0.2338	0.656 (0.110–3.903)	0.6429
Smoke	1.221 (0.534–2.794)	0.6363	4.069 (1.073–15.436)	*0.0391*	0.538 (0.175–1.654)	0.2793
Alcohol	1.192 (0.249–5.691)	0.8261	0.048 (0.001–1.744)	0.0976	11.172 (0.878–142.131)	0.0629
Education	0.473 (0.203–1.099)	0.0818	0.478 (0.108–2.120)	0.3313	0.288 (0.057–1.462)	0.1331
Occupation	1.363 (0.708–2.625)	0.3547	2.231 (0.847–5.876)	0.1043	1.264 (0.371–4.309)	0.7084

Italic values indicate significance of *p* < 0.05

## Discussion

In this study, we adopted IR-induced renal injury model to observe chronic progression of renal tubular injury and interstitial fibrosis. Firstly, we found that KA reduced IR-induced abnormal serum BUN concentration as well as total triglycerides with statistically significant difference, improved renal function and hyperlipidemia in mice. Kidneys play an important role in the protein reabsorption and excretion of protein metabolites. Renal injury causes abnormal protein metabolism, which can affect enzyme-related protein synthesis and metabolism, resulting in hyperlipidemia [25, 26]. KA acted as an essential protein supplement and alleviated hypoproteinemia and protein synthesis in liver, we inferred from our results that KA might increase the expression of lipoprotein lipase, hepatic lipase and very low-density lipoprotein receptor, and as a result increase triglycerides clearance and decrease their formation in liver, thereby modifying the hemostasis of triglyceride. KA also caused a slight but statistically insignificant improvement on total cholesterol compared to the IR group. This might be due to the different metabolic mechanisms involved for cholesterol and triglycerides. Cholesterol biosynthesis is dependent on the enzyme activity of 3-hydroxy-3-methylglutaryl-CoA (HMG-CoA) in liver. In chronic kidney disease both the expression and activity of HMG-CoA showed a marked increase [27]. The reduction of hypercholesterolemia induced by renal injury depends on the inhibition of HMG-CoA, such as statins.

Secondly, we observed an anti-inflammatory and -apoptotic effect of KA in mice. As reported, at the early stage of renal injury, inflammatory response is a protective measure to attenuate injury and promote repair. When inflammatory infiltration is uncontrolled, abundant inflammatory cells recruited from circulation or resident cells accumulate in the tubular interstitium, producing massive amounts of proinflammatory molecules, such as IL-1β, IL-6, tumor necrosis factor-α, and dysregulated renal immunoreaction further deteriorates renal injury [21, 28]. Apoptosis is another cause accounting for renal injury. Proximal tubular epithelial cells are highly susceptible to IR injury, the necrotic or apoptotic tubular cells release harmful molecules, form cell casts, resulting in renal obstruction which further exacerbates renal injury [29]. NF-κB pathway was widely reported as transcription factors participating in renal inflammation and apoptosis by regulating the expression of some pro-inflammatory cytokines and chemokines genes [30–35]. It was shown that the MAPK signaling pathway, which includes ERK and p38 kinase, is activated by inflammatory stimuli and involved in regulating p65 NF-κB activation to promote inflammation [36]. In the present study we observed increased inflammatory T cells and neutrophils infiltration and tubular apoptosis in IR group as well as activation of NF-kB and MAPK signaling pathways. KA administration alleviated inflammatory infiltration of mature T cells and neutrophils in mice. Also, tubular apoptosis caused by IR was attenuated in KA group too, exhibiting the anti-apoptotic effect of KA treatment. Furthermore, NF-kB and MAPK signaling pathways were significantly inhibited in the IR + KA group too, as well as downstream molecule NFATc-1. We inferred that the decrease of inflammation and apoptosis brought by KA administration might have some links with an inhibited activation of NF-κB and MAPK pathways. As MAPK are catalytically inactive in base condition and their activation requires specialized enzyme to phosphorylate their activation loops. KA might inhibit MAPK pathway by the reduction of associated phosphatases. NF-κB is distributed in the cytoplasm and translocates into nucleus when stimulated, KA might inhibit the NF-κB pathway activation by reduction of stimulating factors or the receptor activator of NF-κB, or by the inhibition of MAPK pathway. Further studies to explore the detailed mechanisms of KA need to be done.

In addition, we explored the effect of KA intervention on CKD patients. Although numerous clinical trials on the effect of KA and LPD on CKD were conducted in past years, the results were controversial [37–40]. Some studies found that the potential benefits of KA and LPD were mainly focused on reducing waste products of protein metabolism, such as modification of creatine concentration. However, their influence on renal function protection or alleviating CKD progression required more careful and adequate attention [37, 41–44]. The uncertain effect of protein restriction on CKD protection might be related to the progressive and irreversible characteristic of CKD, especially CKD patients at stages 4 and 5. Most patients recruited in KA and LPD studies were with eGFR less than 30 mL/min, or in a pre-dialysis state [8, 45, 46]. Their worse physical conditions made KA and LPD intervention only an adjunctive therapy to main therapy such as glucocorticoids and immuno-biologicals. In the meantime, different pathological types showed inconsistent progression rates, for example CKD development in patients with membranous nephropathy exacerbated more rapidly than in patients with mesangial proliferative nephritis. Different degrees or types of pathological nephropathies could also influence the results of clinical study. Furthermore, as the study on KA and LPD was conducted over a long time period, often more than 6 months, the compliance of patients recruited was especially important. An unstrict administration of LPD could influence the final result of the clinical study.

Our clinical research collected data from CKD patients from 2015 to 2018 in Tongji hospital, which partially represent the population in the area of central China along the Yangtse River. Among all the CKD patients enrolled, 119 were at CKD stages 1 and 2, only 9 patients were prescribed with KA, compared to 323 at stages 3 to 5, with 148 prescribed with KA. Thus, only CKD patients at stages 3 to 5 were taken into consideration. Our next step was to analyze the renal function between KA and No-KA group. There was a great difference in baseline renal function indexes between KA and No-KA groups in patients at CKD stage 3, patients on KA appeared to be in worse renal conditions. We speculated this may be due to the deep relationship between eGFR and the accumulation of metabolic protein waste. The lower the eGFR was, the less efficiently protein wastes were excreted from our body and the greater the accumulation of waste and toxicities, the more urgent the need for a protein intake intervention. Thus, patients at stages 3 to 5 were more often prescribed KA treatment to reduce renal load than patients at earlier stages. Using early research as reference, we defined a reduction of > 50% eGFR as an end-point event. The patients with dialysis or renal transplantation at the time of enrolment were excluded. According to Kaplan–Meier analysis, the cumulative probability to reach the end-point was lower in the KA group than the No-KA group among patients at stages 4 and 5. Otherwise, according to our data the age of patients at CKD stages 3 to 5 peaked at middle age, mainly between 34 and 60 years old (Table 2). It presented a probable protective effect of KA on CKD middle aged patients at stages 4 and 5, which meant KA was a protective factor in staving CKD progression. Our study implied that a supplement of KA could defer the progression of CKD in patients at stages 4 and 5, which was consistent to the animal experiment results and previous research [2, 47–49].

CKD is a chronic progressive disease which could affect the whole-body system and finally irreversibly enter end-stage kidney stage [50, 51]. Finding a way to slow down the development of chronic renal injury was important for both family and social burdens. This study demonstrated the effect of KA on IR-induced murine CKD, by inhibiting the activation of NF-κB and MAPK pathways, thereby alleviating inflammatory infiltration and apoptosis, finally attenuating renal tubular injury and interstitial fibrosis. Meanwhile, a clinical trial was conducted to demonstrate its effect on delaying CKD progression. There were still some limitations to which we are still determining the best approach: (1) In animal experiments, there was no evidence to prove that the inhibition of NF-κB and MAPK directly attenuated inflammation and apoptosis. As NF-κB and MAPK pathways participate in several physical and pathological processes, inhibitors of these two pathways might be needed to demonstrate the direct relationship between the pathways and inflammation and apoptosis caused by KA. (2) In clinical trials, apart from KA, other medications were not strictly consistent. (3) Human inflammatory and apoptotic factors need to be measured for a further demonstration of anti-inflammatory effect from KA. More longitudinal studies are required to perfectly support the anti-inflammatory and -apoptotic effects of KA and LPD on CKD progression.

## Conclusions

We demonstrated experimental evidence indicating that KA played a protective role in IR induced renal injury and fibrosis, via an inhibition of NF-κB and MAPK pathway to attenuate inflammation and apoptosis. In the meantime, we observed the therapeutic effect of KA on staving CKD progression in the clinical setting, mainly in end-stage patients. Our study provided theoretical support for KA treatment in alleviating the progression of ischemic renal injury into CKD.

## Abbreviations

CKD: chronic kidney disease
LPD: low protein diet
IR: ischemia–reperfusion
KA: compound α-ketoacid tablets
eGFR: estimated glomerular filtration rate
Scr: serum creatine
BUN: blood urea nitrogen
NF-κB: nuclear factor-kappa B
MAPK: mitogen-activated protein kinase
VLPD: very low protein diet
KLF15: Kruppel-like factor-15
LTL: lotus tetragonolobus lectin
Kim-1: kidney injury molecule-1
α-SMA: alpha-smooth muscle actin
PDGFR-β: platelet-derived growth factor receptor
NFATc-1: nuclear factor of activated T cells c1
ERK: extracellular regulated protein kinases
